# Ethical Implications of the Fecal Microbiota Transplantation: Disclosure of a False-Positive HIV Test

**DOI:** 10.1155/2021/6696542

**Published:** 2021-12-06

**Authors:** Caitlyn Hollingshead, Jacob Ciricillo, Joel Kammeyer

**Affiliations:** The University of Toledo, 3000 Arlington AveToledo, OH 43614, USA

## Abstract

Fecal microbiota transplantation (FMT) has gained popularity as an effective therapeutic option for *Clostridioides difficile* infection (CDI). Since its FDA recognition as a treatment modality for recurrent CDI in 2013, screening protocols for FMT donor stool have been in flux. However, extensive health questionnaires, in combination with serological and stool assays, have become mainstays in the donor screening process, although ethical implications are yet to be thoroughly considered. Herein, we present the case of a family member found to have a false-positive HIV test during the donor screening process and discuss potential ethical ramifications associated with FMT stool donation.

## 1. Introduction

Recurrent *Clostridioides difficile* infection (CDI) has established itself as a leading cause of infectious antibiotic-associated diarrhea worldwide, recurring in 20–30% of CDI cases [1]. Due to the increasing incidence of recurrent CDI and its evolving burdens on the healthcare system, fecal microbiota transplantation (FMT) has gained popularity as a therapeutic option for recurrent CDI [2, 3]. A recent meta-analysis exploring the efficacy of FMT, including thirty-seven studies, revealed clinical resolution of infection in 92% of cases of recurrent and refractory CDI [4]. Patients with multiple CDI episodes refractory to antibiotic therapy are candidates to undergo FMT, which has demonstrated decreased CDI-related mortality in hospitalized patients with recurrent CDI [5, 6]. Choosing a family member as a donor can be advantageous due to similarities in microbiota and cost considerations. However, the screening process may present hereunto unconsidered ethical ramifications.

## 2. Case Presentation

A 54-year-old male was identified as an appropriate potential donor for fecal transplantation. His father, hospitalized for a fourth recurrence of *C. difficile* colitis after multiple failed antibiotic courses, was referred for FMT. As part of routine screening to evaluate his suitability as a donor, the son's HIV p24 antigen returned positive. The son was informed of his positive result by medical staff and was referred to infectious diseases without counseling. The son was in a monogamous relationship with his wife and had no risk factors for HIV infection. Confirmatory testing using a Multispot HIV-1 and HIV-2 antibody differentiation assay returned as negative, and subsequent HIV viral load was negative. The son was then counseled that he had a false-positive HIV test by an infectious diseases physician.

## 3. Discussion

**Figure 1:**
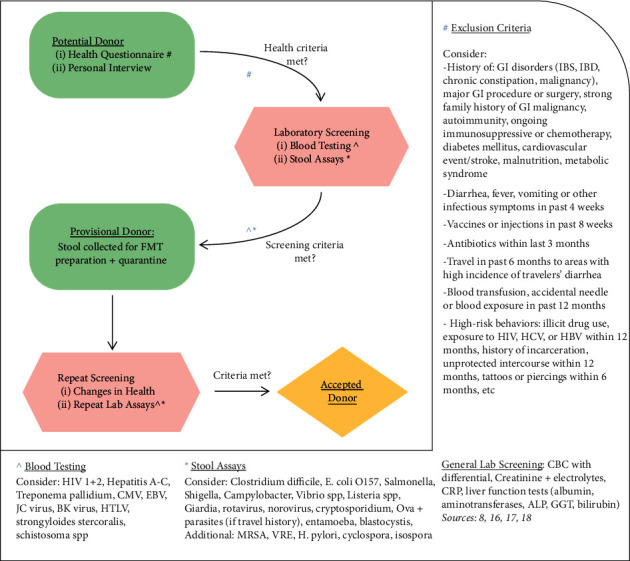
Summary of the FMT donor screening process: including the review of exclusion criteria, blood testing, and stool assays undergone by potential donors prior to authorization as an accepted donor. Note that these criteria are to be used as guidance for donor selection. However, this list of screening assays is not exhaustive, and additional testing may be considered in certain clinical scenarios.

FMT was first utilized as an accepted treatment modality for *C. difficile* colitis in the United States in 2013 when it gained FDA approval. However, it was first utilized for the treatment of pseudomembranous colitis in 1958 [7]. Its first use for a noninfectious cause was noted in 1989 when it was used for a patient with ulcerative colitis [7]. There are no universally accepted screening protocols for stool donors, but European guidelines recommend screening potential donors for HIV, cytomegalovirus, Epstein–Barr virus, hepatitis A, hepatitis B, hepatitis C, hepatitis E, syphilis, and *Entamoeba histolytica* [8]; see also Figure 1.

As FMT is a relatively new treatment modality, new ethical considerations are raised regarding how potential donors with positive screening tests are best handled. In this case, the patient was informed he had HIV without receiving counseling, and further testing revealed this to be a false-positive result. This disclosure imposes consequences, including potential isolation from the spouse or family and significant stigma. Further ethical considerations are raised upon consideration of what would have transpired had the result truly been positive: the physician must inform the father that his son was not a compatible match while protecting the son's autonomy.

Procuring stool from a stool bank from prescreened donors can cost between $1500 and $2000 [9]; therefore, choosing a family member as a donor is an attractive alternative due to decreased cost. Screening any potential donor for transmittable infectious diseases is an important step before donor selection. Several organizations have developed screening recommendations for donors that include HIV screening [10]. However, those ordering testing in this circumstance may not be well versed in counseling after a positive screening test. Current HIV testing has an extremely high specificity; however, in populations of low prevalence, the positive predictive value is extremely low [11]. Those involved in the screening of FMT donors should have procedures in place to provide counseling after positive results.

Many obstacles prevent FMT from being more frequently utilized for CDI [12]. Challenges include donor recruitment and consistent regulation of donor stool screening, with a recent example demonstrating only 3% of candidate donors ultimately qualifying for donation after screening [13]. The utilization of stool banks would have avoided the ethical dilemma in this case. They provide reliable donors and spare time spent undergoing prescreening while avoiding the risk of false-positive tests and the associated psychological harm to their family members. However, there have been recent reports of transmission of enteropathogenic *E. coli* and Shiga toxin-producing *E. coli* via FMT after donor stool was obtained from stool banks [14], which bring into question their screening practices.

Available data reveal excellent short-term safety data with FMT. However, it should be noted that most currently available randomized controlled studies have small sample sizes and short follow-up periods of eight to twelve weeks, making the long-term complications of FMT unknown [15]. In lieu of utilization of a stool bank, robust evidence-based guidelines for the screening of FMT donors are needed, as well as more randomized controlled studies with longer follow-up periods to demonstrate long-term safety data. Additionally, future guidelines regarding the usage of this modality should include guidance for gastroenterologists regarding disclosure and confirmation of any positive results of screening for infectious diseases of potential donor samples.

## Data Availability

We have no underlying data to reference.
